# Structural Insights on PHA Binding Protein PhaP from *Aeromonas hydrophila*

**DOI:** 10.1038/srep39424

**Published:** 2016-12-23

**Authors:** Hongyu Zhao, Hui Wei, Xi Liu, Zhenyu Yao, Manyu Xu, Daixu Wei, Jiawei Wang, Xinquan Wang, Guo-Qiang Chen

**Affiliations:** 1 Center for Synthetic and Systems Biology, School of Life Science, Tsinghua-Peking Center for Life Sciences, Tsinghua University, Beijing 100084, China; 2 Key Laboratory of Cancer Prevention and Therapy, National Clinical Research Center for Cancer, Tianjin Medical University Cancer Institute and Hospital, Tianjin, China; 3 MOE Laboratory of Protein Science, Beijing Advanced Innovation Center for Structural Biology, Collaborative Innovation Center for Biotherapy, School of Life Sciences, Tsinghua University, Beijing 100084, China; 4 Collaborative Innovation Center for Biotherapy, State Key Laboratory of Biotherapy and Cancer Center, West China Hospital, West China Medical School, Sichuan University, Chengdu, China; 5 Center for Nano and Micro-Mechanics, Tsinghua University, Beijing 100084, China; 6 MOE Key Lab for Industrial Biocatalysis, Tsinghua University, Beijing 100084, China

## Abstract

Phasins or PhaPs are a group of amphiphilic proteins that are found attached to the surface of microbial polyhydroxyalkanoate (PHA) granules. They have both structural and regulatory functions and can affect intracellular PHA accumulation and mediate protein folding. The molecular basis for the diverse functions of the PhaPs has not been fully understood due to the lack of the structural knowledge. Here we report the structural and biochemical studies of the PhaP cloned from *Aeromonas hydrophila* (PhaP_*Ah*_), which is utilized in protein and tissue engineering. The crystal structure of PhaP_*Ah*_ was revealed to be a tetramer with 8 α-helices adopting a coiled-coil structure. Each monomer has a hydrophobic and a hydrophilic surface, rendering the surfactant properties of the PhaP_*Ah*_ monomer. Based on the crystal structure, we predicted three key amino acid residues and obtained mutants with enhanced stability and improved emulsification properties. The first PhaP crystal structure, as reported in this study, is an important step towards a mechanistic understanding of how PHA is formed *in vivo* and why PhaP has such unique surfactant properties. At the same time, it will facilitate the study of other PhaP members that may have significant biotechnological potential as bio-surfactants and amphipathic coatings.

Polyhydroxyalkanoates (PHA) are accumulated as insoluble granules in the bacterial cytoplasm, where they serve as intracellular carbon and energy sources^1^,^2^. PHAs have been extensively studied as biopolyesters with various useful properties including thermo-processability, biodegradability, biocompatibility and even piezoelectricity^3^,^4^. These properties make PHAs strong candidates for novel, environmentally friendly bioplastics^5^,^6^. PHA granules also allow bacteria to survive under conditions of biotic and abiotic stress, such as temperature shifts, pH and osmotic pressure, or lack of nutrients^7^,^8^. PHAs have even been exploited as implant biomaterials and drug delivery matrices^7^,^8^,^9^,^10^.

PHAs are stored intracellularly as granules surrounded by a layer of phospholipids and several types of granule-associated proteins including PHA synthases, PHA depolymerases and a group of proteins known as phasins or PhaPs^11^. PhaPs are proteins that bind to the surface of PHA granules, and can be regarded as an interphase between the cytoplasm and the hydrophobic PHA granules which functions in preventing granular coalescence^12^. Studies of PhaPs have opened up a broad range of potential applications in biotechnology and medicine, from protein purification to targeted drug delivery^13^,^14^,^15^. In addition to their structural role on the surface of PHA granules, other PhaP functions have been reported as well. PhaPs can influence the number and sizes of PHA granules and promote PHA accumulation^12^,^16^. Some PhaPs were reported to have regulatory activity, such as PhaF from *Pseudomonas putida*, which regulates PHA synthesis^17^, and ApdA of *Rhodospirillum rubrum*, which activates polyhydroxybutyrate (PHB) depolymerization^18^. Effects of PhaPs on PHA accumulation are due to several possible reasons, including the activation of genes involved in PHA synthesis^19^,^20^,^21^ and the passive formation of a barrier between the PHA granules and other intracellular components which might in turn help avoid negative effects of PHA accumulation on cell physiology^12^. PhaP from *Azotobacter sp.* FA-8 was demonstrated to not only improve growth and PHB accumulation in recombinant *E. coli*, but also to protect non-PHB synthesizing *E. coli* under both normal and stress conditions, leading to higher growth and resistance to both heat shock and superoxide stress^12^. This type of PhaP was also shown to affect protein folding in a chaperone-like manner both *in vivo* and *in vitro*^22^.

PhaP_*Ah*_ isolated from *Aeromonas hydrophila* strain 4AK4 was observed to affect the PHA polymerase at different levels^19^. It has been used as a specificity tag for binding to PHA particles for protein purification and drug delivery as well as other applications^14^,^15^,^23^. The PhaP-based low-cost protein purification system was successfully demonstrated in the purification of enhanced green fluorescent protein (EGFP), maltase binding protein and β-galactosidase^14^,^15^,^23^. Additionally, the growth of mammalian cells was found to be strongly supported by biopolymers coated with PhaP_*Ah*_–RGD^24^, while a drug targeting system based on PhaP_*Ah*_ fused with targeted cell ligands has been reported to achieve specific binding to cancer cells^25^. Finally, PhaP_*Ah*_ has been applied as a highly efficient protein surfactant^26^.

However, although PhaP has these remarkable properties, no studies have been reported on its structure other than theoretical predictions^22^. This is all the more remarkable since PhaP_*Ah*_ belongs to the phasin 2 family, which is the most numerous one among 952 sequences known^27^. In this study, the crystal structure of PhaP_*Ah*_ has been revealed^28^, and a mechanism proposed for its binding to hydrophobic PHA materials. Finally, the protein’s stability and surfactant properties have been successfully enhanced by rational mutagenesis, fully establishing the crystal structure’s biological relevance.

## Results

### Overall crystal structure of PhaP_*Ah*_

The PhaP_*Ah*_ protein (residues 1–116) was successfully expressed in *E. coli* BL21 (DE3), and was further purified using size-exclusion chromatography (Fig. S1). PhaP_*Ah*_ native crystals diffracted to 3.0 Å, as observed using the Shanghai Synchrotron Research Facility (SSRF) BL17U beamline. They belonged to the *C*222_1_ space group with cell dimensions of a = 131.78 Å, b = 174.14 Å and c = 198.82 Å. Initial experimental phases were solved using the single-wavelength anomalous diffraction (SAD) method with a (Ta_6_Br_12_)^2+^ cluster soaked PhaP_*Ah*_ crystal that diffracted to 4.0 Å.

**Figure 1:**
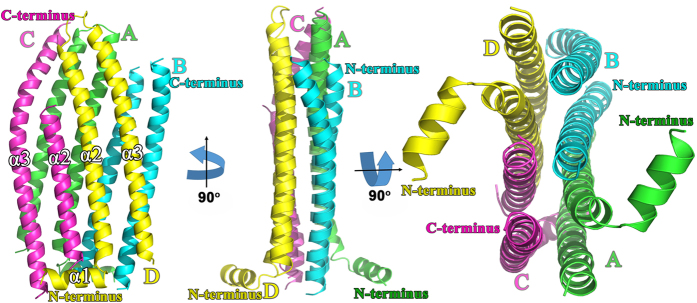
Overall crystal structure of PhaP_*Ah*_. Crystal structure of a PhaP_*Ah*_ tetramer consisting of four chains designated A (green), B (cyan), C (purple) and D (yellow), respectively. Chains A and D consists of α1, α2 and α3 helices and the linkers connecting them. Chains and B and C adopt a different conformation. The final models of these two monomers include α-helices α2 and α3, and the α1 helix and the linkers were not built due to weak densities in these regions. The N- and C-terminus of each chain is indicated. The figure shows three different view of the same structure.

After rounds of phase improvement and extension, model building, and refinement, the final structure was refined to a resolution of 3.00 Å with R_*work*_ of 22.70% and R_*free*_ of 29.50% (Table 1). Size-exclusion chromatography and dynamic light scattering (DLS) analyses indicated that PhaP_*Ah*_ exists as a tetramer in solution (Figs S1 and S2) and the final model in the asymmetric unit consists of four PhaP_*Ah*_ tetramers accordingly (Fig. S4). The overall structure of a tetramer consists of residues M3 to I109 from chain A, F9 to N51 and T62 to A106 from chain B, A17 to L50 and Q60 to G114 from chain C, as well as M3 to K110 from chain D (Fig. 1). The overall shape of the tetramer somewhat resembles a flat rectangular brick, with two short N-terminal helices from chains A and D protruding outwards from two opposite sides of the “brick”. Chains A and D have two long helices after the short N-terminal helix 1 (residues 17–60 for helix 2 and 66–109 for helix 3) that are connected by a short linker (residues 61–65). The built models of chains B and C are less complete with only helices 2 and 3 (residues 9–51 and 62–106 of chain B, residues 17–50 and 60–114 of chain C) fully resolved.

The conformation of chains A and D is different from chains B and C, and will be described in more detail in the following section. The two protruding helices from two monomers in the tetramer are responsible for the continuous stacking of tetramers in a helical manner along one direction in the crystal structure (Fig. S4). At the interface of the ABCD tetramer with the above EFGH and down IJKL tetramers, the protruding helices from chains A and D interact with chain G from the EFGH tetramer and chain K from the IJKL tetramer, respectively (Fig. S4).

### Two conformations of the PhaP_*Ah*_ monomer

**Figure 2:**
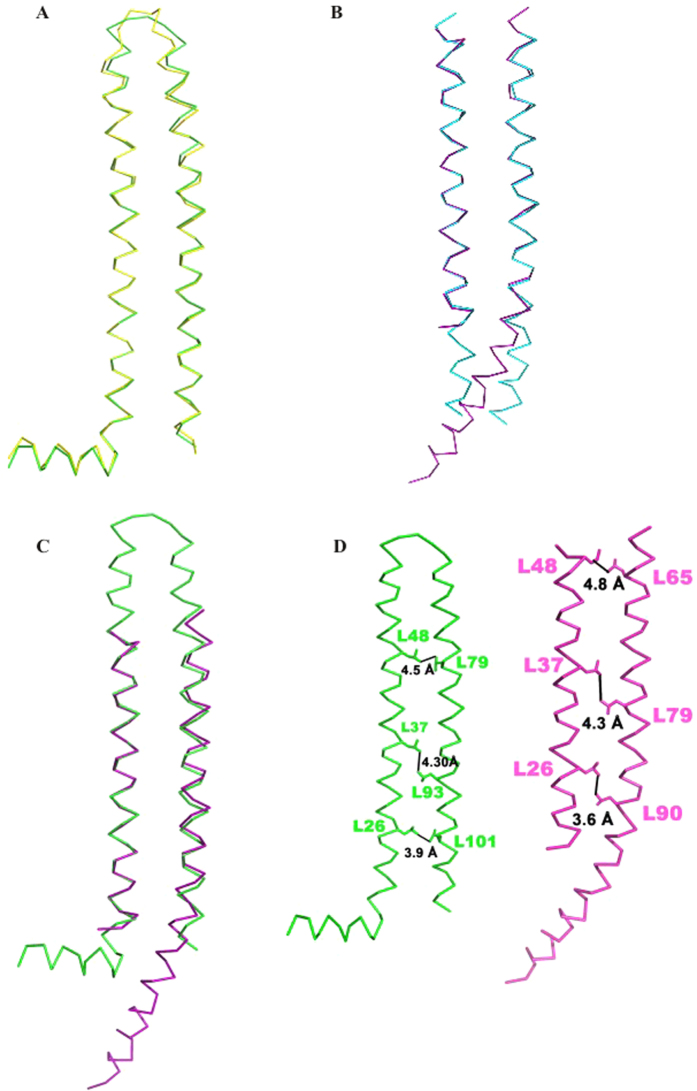
Two different conformations of a PhaP_*Ah*_ monomer. (**A**) Superimposed chain A (green) and chain D (yellow). (**B**) Superimposed chain B (cyan) and chain C (purple). (**C**) Superimposed chain A (green) and chain C (purple). (**D**) The inter-chain hydrophobic interaction of chain A (green) and chain C (purple) indicated by black linking lines.

The four monomers in the PhaP_*Ah*_ tetramer adopt two distinct conformations: type I, observed in chains A and D and type II, observed in chains B and C (Fig. 2A,B). The type I chains A and D are structurally conserved with an r.m.s.d of 0.78 Å for 106 Cα atoms after superimposition. The type II, B and C chains are less conserved with an r.m.s.d of 2.2 Å for only 48 Cα atoms after superimposition, with the largest deviation at the C-terminal end of helix 3. When chains A and C are superimposed based on helix 2, a major conformational difference is found between types I and II, in which the position of the entire helix 3 shifts downwards in chain C (Fig. 2C). The residues L26, L37 and L48 have hydrophobic interactions with L101, L93 and L79, respectively, in chain A, whereas the interacting residues in chain C change to L90, L79 and L65, respectively, due to the positional shift of helix 3 (Fig. 2D).

### Structural basis for the emulsification of PhaP_*Ah*_ monomer and a proposed assembly pathway of the tetramer

**Figure 3:**
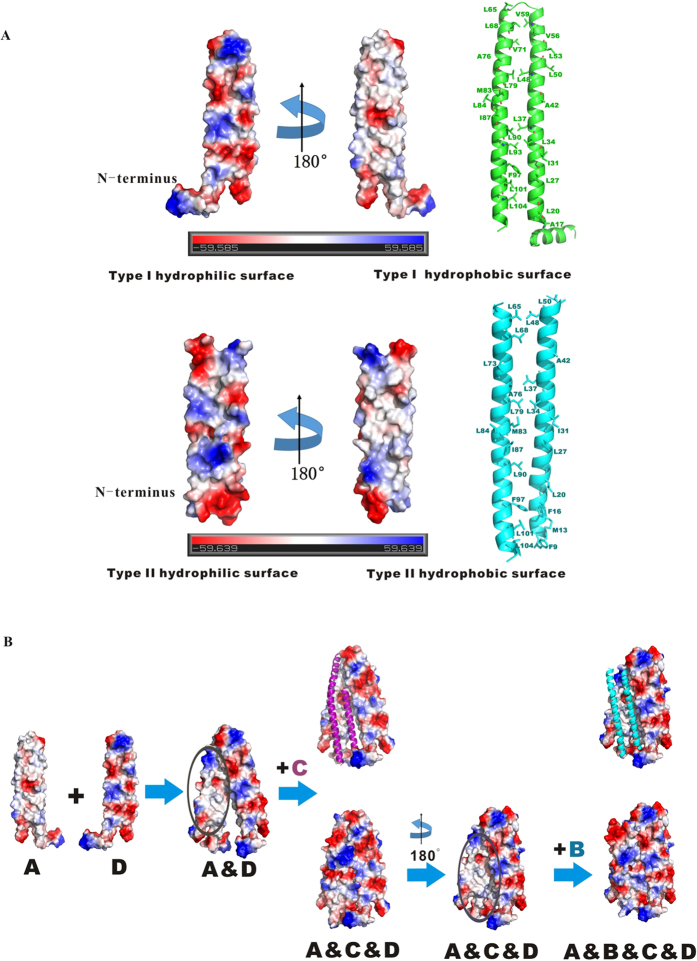
Mechanistic explanation of surfactant properties of PhaP_*Ah*_ and hypothetical formation process of a PhaP_*Ah*_tetramer. (**A**) The amphiphilic surfaces of type I and II chains. Type I is found in chains A and D, and type II in chains B and C. All hydrophobic amino acid side chains are shown for both types. (**B**) The simulated process of tetramer formation via hydrophobic interaction. It begins from a dimer of chains A and D, chains C and B follow.

Previous studies demonstrated the surfactant properties of PhaP_*Ah*_^26^. As PhaP is able to bind to exposed hydrophobic surfaces in the cell, such as PHA granules, it also shows chaperone-like properties^29^. Surface electrostatic potential analysis was conducted on type I (A and D) and II (B and C) chain conformations of the monomer. A very significant phenomenon, observed in both conformations, was the focused distribution of hydrophilic and hydrophobic residues on two opposite sides of the PhaP_*Ah*_ monomer (Fig. 3A). Therefore, a PhaP_*Ah*_ monomer is a naturally amphiphilic polypeptide. The residues contributing to the hydrophobic and hydrophilic surfaces in the type I and II conformation have been listed (Table 2). Thus, the intrinsic amphiphilic structure of PhaP_*Ah*_ explains its surfactant properties.

The formation of the tetramer can accordingly be rationalized the following way (Fig. 3B): chains A and D expose their respective hydrophobic surfaces to each other, while loop regions of these two chains are next to each other forming a shape resembling chopsticks. The still exposed hydrophobic regions of the stacked A–D dimer then recruit chains B and C through hydrophobic interactions to form the tetramer. The recruitment of chains B and C leaves no hydrophobic region exposed to the solvent environment in the tetramer (Fig. 3B). This simulated assembly pathway explains that PhaP_*Ah*_ exist as stable tetramers in solution. It also partially clarifies how chains B and C might be distorted to fit chains A and D through their exposed hydrophobic regions, since this distortion can be promoted and stabilized by the hydrophobic interaction.

### Rational mutagenesis for improved stability and surfactant properties

**Figure 4:**
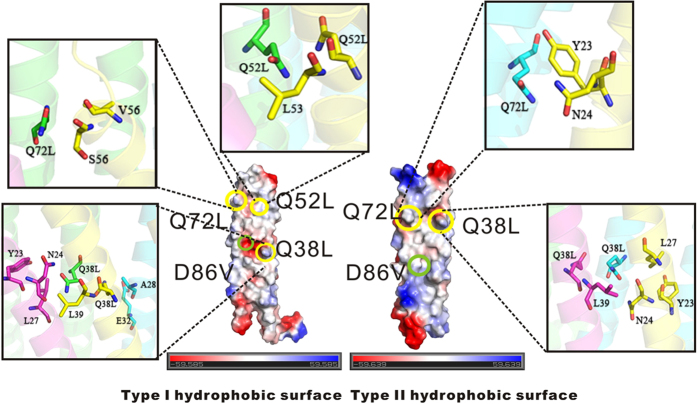
Specific Point mutations on PhaP_*Ah*_. Residues of PhaP_*Ah*_ were chosen for mutagenesis based on the presence of hydrophilic amino acids within a predominantly hydrophobic surface. Mutants Q38L, Q52L, Q72L and D86V were designed to investigate the variation of surfactant properties. In chain A, which has a type I hydrophobic surface, the mutant Q38L can form hydrophobic interactions with Y23, N24 and L27 from chain C, mutant Q38L and the wild-type residue L39 from chain D, as well as A28 and E32 from chain B. Mutant Q52L can form hydrophobic interactions with mutant Q52L and the wild-type residue L53 from chain D. The mutant Q72L can form hydrophobic interactions with residues V56 and S57 from chain D. Chain D also has a type I surface. While the mutant Q38L in chain B forms hydrophobic interactions with Y23, N24 and L27 from chain D and mutants Q38L and L39 from chain C, the mutant Q72L in chain B forms hydrophobic interactions with residues Y23 and N24 from chain D. The chains B and C both have a type II surface.

Based on PhaP_*Ah*_ structure information, four hydrophilic amino acids embedded in the otherwise hydrophobic interaction surface were selected as targets for rational mutations that may improve amphiphilic properties (Fig. 4). Thus, the hydrophilic glutamines Q38, Q52 and Q72 were mutated to hydrophobic leucines and the mutant proteins were successfully expressed and purified. The mutant D86V failed to be expressed, implying a likely important role for the negatively charged core containing D86.

Judging by the detailed structural information available (Fig. 4), one can thus surmise that Q38L from chain A can form hydrophobic interactions with Y23, N24 and L27 from chain C, Q38L and L39 from chain D, as well as A28 and E32 from chain B. Q38L from chain B undergoes hydrophobic interactions with Y23, N24 and L27 from chain D, as well as Q38L and L39 from chain C. Chain D is analogous to chain A, since both of them have a type I surface, whereas chain C is analogous to chain B, as both have a type II surface. As a result, the stability of the tetramer can be improved by the Q38L mutation because of the improved hydrophobic interactions between all four chains.

Similarly, the Q52L and Q72L mutations would also enhance the stability of the tetramer by increasing hydrophobic interactions.Q52L from chain A can undergo hydrophobic interactions with Q52L and L53 from chain D, whereas Q72L from chain A can interact with V56 and S57 from chain D. While the two mutations in the type I hydrophobic surface can enhance the hydrophobic interactions between these two type I chains, Q72L from chain B can form hydrophobic interactions with Y23 and N24 from chain D, indicating that Q72L in the type II hydrophobic surface can enhance the hydrophobic interactions between type I and II chains.

Above all, the most important reason for choosing these three mutations was to improve the stability of PhaP_*Ah.*_. Another reason was to enhance its emulsification abilities as a result of introducing additional hydrophobic amino acids into the *bona fide* hydrophobic surface.

### Rational mutations of PhaP_*Ah*_ resulted in stability improvement

**Figure 5:**
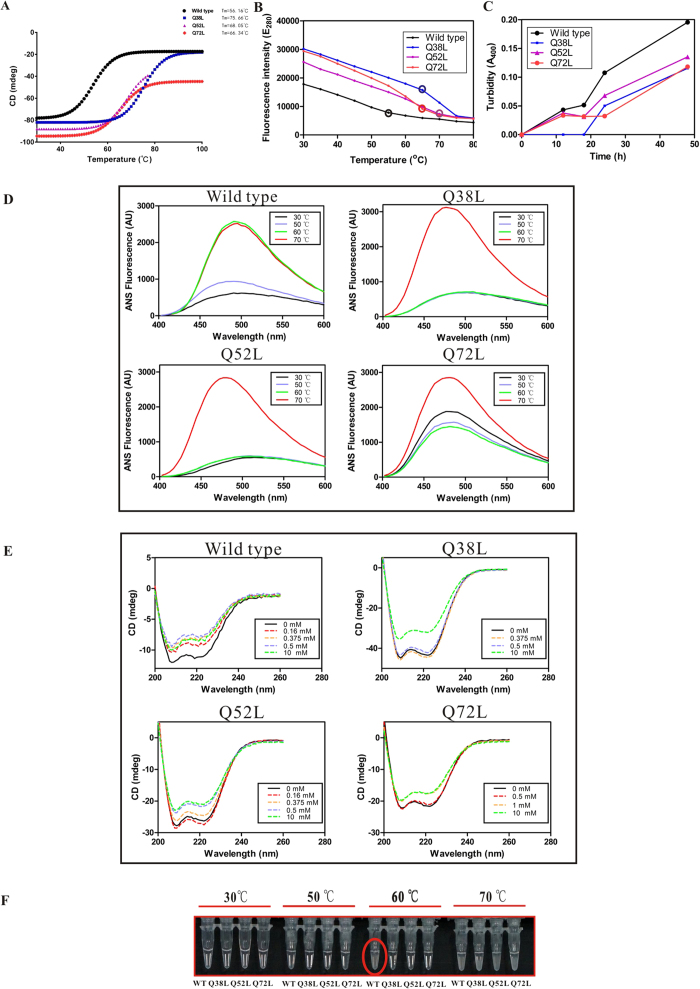
Change in PhaP_*Ah*_ stability caused by specific point mutations. (**A**) Melting temperatures of PhaP_*Ah*_ wild type and mutants studied by CD. Thermal graphs were recorded between 30 °C to 100 °C. The following T_m_ values were obtained: wild type (56.16 °C), Q38L (75.66 °C), Q52L (68.05 °C), Q72L (66.34 °C). (**B**) Temperature-induced denaturation monitored via maximum emission wavelengths of Tyr fluorescence (E_max_). (**C**) Improved secondary structure stability of PhaP_*Ah*_ glutamine to leucine mutants detected by turbidity measurements. PhaP_*Ah*_ protein (0.1 mg/ml) aggregation in an aqueous solution at 37 °C monitored by increase of optical density at 400 nm. (**D**) Hydrophobic exposure of wild-type and mutant PhaP_*Ah*_ proteins at different temperatures studied using ANS fluorescence. (**E**) Improved secondary structure stability of PhaP_*Ah*_ glutamine to leucine mutants measured by CD spectroscopy. CD Spectra of wild type and mutant PhaP_*Ah*_ (0.1 mg/ml) in emulsions ranging from 0 to 10 mM sodium oleate in water. (**F**) Incubation under different temperatures for 30 minutes. All studies were performed using 2.0 mg/mL PhaP_*Ah*_ in buffer containing 50 mM Tris (pH 8.0) and 500 mM NaCl.

Protein melting temperatures T_m_ were compared using circular dichroism (CD) spectroscopy with identical sample concentrations (Fig. 5A, S7). All three mutants showed a higher T_m_ than the wild type, with more than 10 degree increases for Q52L and Q72L, as well as an even more striking 20 degree increase for Q38L. The T_m_ increases clearly demonstrated that an improvement of thermal stability resulted from these three mutations, most likely due to a tighter hydrophobic interaction in the tetramer core. Furthermore, the study of 1-anilinonaphthalene-8-sulfonate (ANS) binding affinity also showed consistent results. In the wild-type, exposure of hydrophobic sites was evident already at 50 °C, with a large increase at 60 °C, without any further change at 70 °C, suggesting complete melting at 60 °C. The mutants Q38L and Q52L, on the other hand, did not show any observable differences between 30, 50 and 60 °C, followed by what appears to be rapid and complete melting at 70 °C. The mutant Q72L showed an intermediate result, with an inconsistency at 30 °C (Fig. 5D).Additionally, the maximum emission wavelength of intrinsic Tyr fluorescence E_max_ was measured at different temperatures. A sharp transition was observed at 55 °C for the wild-type protein, while transition temperatures of 65 °C, 70 °C and 65 °C were measured for mutants Q38L, Q52L and Q72L, respectively (Fig. 5B). Finally, simple heat incubation studies with visual inspection can also provide valuable evidence of improved thermal stability. Solutions of wild-type protein became turbid and aggregated after incubation at 60 °C, whereas all three mutants remained soluble under the same conditions (Fig. 5F). Taken together, multiple sets of data show what appears to be a clear increase in temperature stability of the *bona fide* hydrophobic core stemming from the addition of hydrophobic residues.

As PhaPs are normally found embedded in the lipid-covered surface of PHA granules, PhaP structure could be affected by hydrophobic environments. Sodium oleate is commonly used as a hydrophobic mimic of PHA granules^30^, and PhaP from *Azotobacter sp*. FA8 (PhaP_Az_) was already observed to change its secondary conformation in the presence of sodium oleate^31^,^32^. It was also suggested that PhaP is able to change its conformation in a hydrophobic environment. It is thus reasonable to assume that PhaP_*Ah*_ behaves in the same way, since it has no obvious hydrophobic regions exposed to the solvent in its soluble tetrameric form (Fig. 3B). PhaP_*Ah*_ thus needs to be able to change its structure and expose its hydrophobic surface in contact with a hydrophobic mimic of PHA. As is shown in Fig. 5E, the secondary structure of wild-type PhaP_*Ah*_ indeed changed in the presence of 0.16 mM sodium oleate, as expected. When the concentration of sodium oleate was further increased to 10 mM, the secondary structure remained the same. On the other hand, the secondary structures of mutants Q38L and Q72L stayed the same in the presence of 0.5 mM sodium oleate, whereas the mutant Q52L began to change its conformation in the presence of 0.375 mM sodium oleate (Fig. 5E). These results suggested that the PhaP_*Ah*_ mutants were able to tolerate higher sodium oleate concentrations than the wild type, indicating that the targeted mutations improved the conformational stability in hydrophobic environments. The reason why the α-helix percentage decreased in the presence of sodium oleate may be attributed to the disruption of the PhaP_*Ah*_ tetramer structure. As is shown in Fig. 3B, chains B and C were distorted to fit chains A and D via their exposed hydrophobic regions. The flexibility of each chain may increase after tetramer structure disruption, possibly leading to the reduction of α-helix percentage.

PhaP_*Ah*_ is a bio-surfactant soluble at ambient temperature in water without any dissolved salts. However, its stability under these circumstance may be compromised. PhaP_*Ah*_ wild-type and the indicated mutants dissolved in water at identical concentration showed increasing turbidity at OD_400_ over a period of 48 h at room temperature, whereby the mutants Q38L, Q52L and Q72L showed a much slower increase of turbidity than the wild-type. All the mutants showed greater stability in their emulsification working environments than that of the wild type (Fig. 5C).

### Targeted PhaP_*Ah*_ mutations improved the protein’s emulsification properties

**Figure 6:**
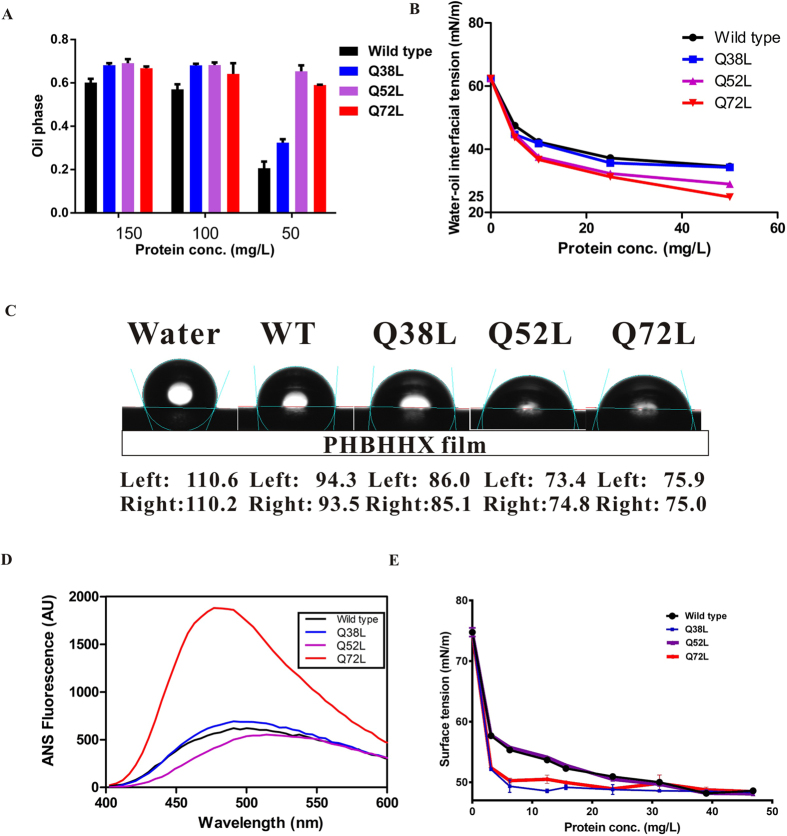
Detection of improved emulsifier properties of PhaPAh glutamine to leucine mutants by multiple methods. (**A**) Emulsibility of oils by various concentrations of wild type and the indicated mutants of PhaP_*Ah*_. Error bars represent standard error of the mean (SEM) from 3 separate competition assays. (**B**) Water-oil interfacial tension in the presence of various concentrations of wild-type and the indicated mutants of PhaP_*Ah*_. (**C**) Comparison of contact angles of drops of 25 mg/L protein solutions of wild-type PhaP_*Ah*_ and the indicated mutants on hydrophobic PHBHHx films. (**D**) Hydrophobic exposure of PhaP_*Ah*_ wild-type and the indicated mutants at 30 °C detected by ANS fluorescence. (**E**) Surface tension of solutions of PhaP_*Ah*_ wild-type and the indicated mutants as a function of protein concentration. Error bars represent standard error of the mean (SEM) from 3 separate measurements.

Emulsification abilities of wild-type PhaP_*Ah*_and the mutants were studied in relation to protein concentration (Fig. 6A). At protein concentrations above 100 mg/L, the emulsification abilities of the mutants were only slightly higher than those of the wild type. Significant differences could be observed as the protein concentration dropped to 50 mg/L, whereby the wild-type PhaP_*Ah*_ did not behave like a surfactant, while the mutants Q52L and Q72L maintained the same emulsification abilities as observed at 100 mg/L. The mutant Q38L presented higher emulsification abilities than the wild type, but it also decreased its emulsification ability at lower concentrations compared to emulsification at 100 mg/L.

Emulsification activity is dictated by the ability of a compound to reduce water-oil interfacial tension. The mutants Q52L and Q72L showed better properties in reducing water-oil interfacial tension than both the wild-type and mutant Q38L (Fig. 6B), which was consistent with the emulsion experiments. Even though the mutant Q38L had the same ability to reduce water-oil interfacial tension as the wild type, it was observed to be more stable (Fig. 5C). As a result, the mutant Q38L can be regarded as a better surfactant than the wild type, while not as good as the mutants Q52L and Q72L due to weaker reduction of interfacial tension. On hydrophobic PHBHHX films, droplets containing PhaP_*Ah*_mutants Q52L and Q72L had smaller contact angles than droplets with the mutant Q38L or wild-type (Fig. 6C), indicating better PHA binding ability.

It can thus be concluded that the two rational mutations Q52L and Q72L, based on our PhaP_*Ah*_ crystal structure, improved the emulsification abilities of PhaP_*Ah*_ allowing lower concentrations of the mutant proteins to achieve the same surfactant effect as much higher concentrations of the wild-type. The different mutated positions led to different PHA binding abilities.

The ability to reduce surface tension (water-air) is another feature of amphipathic molecules. However, since PhaP_*Ah*_ forms tetramers with eight α-helixes in aqueous solutions, it would have to change its conformational structure when exposed to hydrophobic substrates such as PHA. Thus, changes in surface tension were studied in order to compare the differences of tetrameric rather than monomeric hydrophobic sites. The mutant Q72L showed significantly higher 1-anilinonaphthalene-8-sulfonate (ANS) fluorescence than the others, whereas Q38L showed slightly more fluorescence than the wild-type (Fig. 6D). On the other hand, solutions containing the mutants Q38L or Q72L were found to have significantly reduced surface tension compared to solutions containing the wild-type protein at identical concentrations (Fig. 6E).

The surface tension of aqueous solutions containing the mutants Q38L or Q72L was reduced by 10 nN/m compared with the-wild type at a concentration of 10 mg/L (Fig. 6E). The ANS fluorescence assay and surface tension studies showed a consistently increasing trend of tetrameric hydrophobic surface tension. However, the tetramer of mutant Q52L showed the same level of amphipathicity as the wild-type. At concentrations higher than 40 mg/L, all PhaP_*Ah*_ forms reached the critical micelle concentration (CMC) for the tetramer and thus produced the same surface tension. The surface tension study and ANS fluorescence assays further confirmed that the tetramers of the mutants had changed their structure. The mutant proteins also showed more Tyr fluorescence than the wild-type at the same concentration and 30 °C, which is another indication of changed structure of the PhaP_*Ah*_ mutants (Fig. 5B). This further implies changes in the stability of the hydrophobic core, since all the tyrosines are found there (Fig. S6), so that the Tyr residues could have higher intrinsic fluorescence only if the structure of the tetramer has changed (Fig. 5B).

## Discussion

In this work, a high resolution structure of PhaP_*Ah*_ has been successfully obtained for the first time. This allows us to understand the surfactant properties and molecular mechanisms related to the amphiphilic nature of PhaP_*Ah*_. Generally speaking, PhaP_*Ah*_ forms tetramers with 8 α-helixes in aqueous solution and two different types of peptide chains were observed in these tetramers (Fig. 1), whereby a hydrophobic and a hydrophilic surface is present in each monomer (Fig. 3A). When self-assembly of the tetramer occurs in aqueous solution, the hydrophobic surface of each monomer is buried inside the tetramer and so avoids exposure to the aqueous phase (Fig. 3B).

The mechanism by which the tetramer can disassemble into monomers in contact with hydrophobic surfaces, such as PHA granules in the cytoplasm, is also of great interest. An *in vitro* dynamic light scattering (DLS) study^22^ has provided evidence to support this hypothesis, since a monomer size signal from the PhaP_*Ah*_ tetramer was visible via DLS when a hydrophobic phase consisting of 10 mM oleate was added to the Tris-buffer (Fig. S2).

Three specific point-mutations were rationally designed based on the PhaP_*Ah*_ crystal structure. In detail, three hydrophilic glutamines were mutated to hydrophobic leucines, yielding the mutants Q38L, Q52L and Q72L. It was estimated that the mutations should improve the stability of PhaP_*Ah*_ by improving hydrophobic interactions in the tetramer core. Melting temperatures of the mutants were increased, demonstrating enhanced thermal stability of the mutated PhaP_*Ah*_ (Fig. 5A). At the same time, the mutants have shown stronger tetramer stability and emulsification ability under conditions identical to those of the wild-type. The mutants Q52L and Q72L could improve emulsification ability compared to the wild-type because they had better PHA binding ability, whereas the mutant Q38L did not show this improved property.

The tetrameric forms of Q38L and Q72L had more hydrophobic sites than either the wild-type or the Q52L mutant, which may be the result of differences in mutation position and amino acid microenvironment. The mutants also displayed stronger Tyr fluorescence than the wild-type at 30 °C, at the same protein concentration, which offers further evidence of different structural conditions (Fig. 5B). It is interesting to follow and show how the mutations led to structural changes in details. After all, the three PhaP_*Ah*_mutants Q38L, Q52L and Q72L were all improved regarding their stability, which is beneficial for applications in the surfactant industry. At the same time, the mutants Q52L and Q72L showed stronger emulsification ability, which can further improve their application value.

The first 15 amino acids in the N-terminal tail of the type I chains can be observed to stretch out and contact another tetramer (Fig. S4), which may have been useful for crystallization as evidenced by the failure of crystal formation when the N-terminus was truncated. The truncated protein was expressed in *E. coli* and purified using the same purification protocol as the wild-type (Fig. S1). The molecular weight of the truncated PhaP_*Ah*_ protein was found to be about 5 kDa less than that of the wild-type, as revealed by Static Light Scattering (SLS) (Fig. S3), which is consistent with the size reduction.

Interestingly, the emulsification ability of the N-terminally truncated PhaP_*Ah*_was observed to be approximately the same as that of the wild-type (data not shown). PhaP_*Ah*_ has been reported to be a transcription factor of PhaC_*Ah*_^19^, so it should have a DNA-binding region comprising positively charged residues. Based on the crystal structure of PhaC_*Ah*_, the four N-terminal tails can be folded to generate a positively charged groove (Fig. S5). It was demonstrated that a single mutant (D4N) of PhaP_*Ac*_, which has a completely identical sequence to PhaP_*Ah*_, led to increased PHA accumulation in *E. coli* harboring the pha*PCJ* operon from *Aeromonas caviae*^31^. It was hypothesized that replacing the negatively charged aspartate with an asparagine, which is without charge, changed the DNA binding ability. This mutation may promote the interaction between PhaP_*Ac*_ and DNA if the N-terminal tail is the key domain which interacts with DNA. This, in turn, would make the N-terminus dispensable for the surfactant properties, explaining the results seen with the N-terminally truncated protein.

Similarly, with increased understanding of the unique PhaP_*Ah*_ structure, more mutations aimed at improving various PhaP_*Ah*_ properties for various applications can be designed and studied. For example, most PhaPs have been reported to be able to regulate the size of PHA granules and the level of PHA accumulation^12^,^16^. PhaP can also function as a chaperone for better protein folding^29^, or be used as a fusion partner that mediates specific drug targeting and even for protein purifications^13^,^14^. The PhaP_*Ah*_ crystal structure will guide us to find the right mutation locations to design improved phasins for the applications mentioned above. Finally, studies on PhaP as an amphiphilic protein may be able to help us understand the features of other proteins which bind to PHA granules, including their stability, binding and emulsion ability or catalytic properties, as well as inspiring researchers to solve their crystal structures.

## Materials and Methods

### PhaP_*Ah*_ Protein Expression and Purification

The PhaP_*Ah*_ (UniProtKB-O32470) gene was cloned into vector pGEX-6P-1 and expressed as a fusion protein with N-terminal GST tag. The fusion protein was expressed in *Escherichia coli* BL21 (DE3) induced with 0.5 mM isopropyl-β-D-thiogalactopyranoside (IPTG) at 0.6–0.8 OD_600_ in overnight cultures grown at 37 °C. The cells were harvested by centrifugation at 4,000 × g for 15 min and suspended in lysis buffer containing 50 mM Tris (pH 8.0) and 500 mM NaCl. After sonication, the lysates were centrifuged at 13,000 rpm for 1 h, and the supernatants containing GST–PhaP_*Ah*_ transferred to a Glutathione Sepharose 4B column. After extensive washing of the column with lysis buffer, the bound fusion protein was digested on the column with 3 C PreScission protease. The released PhaP_*Ah*_ without the GST tag was collected and further purified by gel-filtration chromatography using a Superdex 200 high performance column (GE Healthcare) with running buffer containing 50 mM Tris (pH 8.0) and 500 mM NaCl. Fractions containing PhaP_*Ah*_ were collected and applied directly to a pre-equilibrated RESOURCE Q column (GE Healthcare) and eluted with a 50–1,000 mM NaCl gradient in 50 mM Tris buffer (pH 8.0). Fractions containing PhaP_*Ah*_ were finally purified using another gel filtration step on the Superdex 200 column same as above. The injected volume was 1 ml with a flow rate of 0.5 ml/min. The same protocol was applied to the mutated proteins.

Selenomethionine (Se-Met) -labelled PhaP_*Ah*_ was expressed using the same plasmid as the native PhaP_*Ah*_. The expression plasmid was introduced into the *Escherichia coli* methionine auxotroph B834 (DE3). An overnight culture was grown in LeMaster media^33^ enriched with 10% LB media to ensure fast growth of the cells. The Se-Met PhaP_*Ah*_ was expressed and purified in a manner similar to the native PhaP_*Ah*_ except LeMaster media was used to guarantee complete substitution of the methionine with selenomethionine.

### Crystallization and Data Collection

The PhaP_*Ah*_ crystals were grown at 291 K under vapor diffusion in hanging drops composed of equal volumes of protein solution [15 mg/ml in 50 mM Tris (pH 8.0) and 500 mM NaCl] and reservoir solution [0.5 M sodium acetate trihydrate, 0.15 M cadmium sulfate hydrate and 0.1 M HEPES buffer (pH 7.5)]^28^. Selenomethionine-labeled crystals were grown under the same conditions. For single wavelength anomalous dispersion (SAD) data collection, crystals were soaked in the wells in a 5 mM (Ta_6_Br_12_)^2+^ solution for 24 h before data collection. All crystals were cryoprotected by soaking in well solution supplemented with 30% (vol/vol) glycerol for 5 s and flash-cooled to 100 K in liquid nitrogen. All datasets were collected on beamline BL17U at the Shanghai Synchrotron Research Facility (SSRF) and processed with HKL2000^32^,^34^.

### Structure Determination and Refinement

The PhaP_*Ah*_ structure was solved using the (Ta_6_Br_12_)^2+^-SAD method. The positions of the Ta atoms were determined using the program SHELXD^35^ and initial phases computed using the program SHELXE^36^ as part of the HKL2MAP package^37^. Density modification was conducted using DM from the CCP4 suite^38^. The resulting electron density map was used for automatic chain building. A data set was collected on the selenomethionine-labeled PhaP_*Ah*_ crystals at the selenium peak wavelength (0.9788 A°), and Se atoms were placed in the asymmetric unit. Based on the density map from the (Ta_6_Br_12_)^2+^-SAD method and the Se-Met signal, automatic model building was performed using the program Arp/wARP^39^. Final model adjustments with the addition of the cadmium cations and structural refinements were conducted using the programs COOT^40^, and PHENIX^41^, respectively. For the final model, the R_*work*_ was 0.2270, and the R_*free*_ was 0.2950. All figures representing structures were rendered using PYMOL ( http://www.pymol.org).

### Emulsion Layer Measurements and Emulsion Index Calculations

0.5 ml aliquots of aqueous solutions containing the wild-type or mutant PhaP_*Ah*_ as water layers with different protein concentrations, were each mixed with 0.5 ml of soybean oil in cylindrical glass vials. The oil-in-water emulsions were then prepared by vortexing (QT-2, Qite, Shanghai, China) at maximum speed for 120 s^26^. Subsequently, the samples were stored in the dark at 25 °C for two days. By measuring the height of the emulsion layer and the total height of the mixed liquid, the emulsion index was calculated according to the following equation^26^:


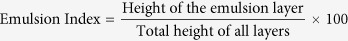


Three parallel samples were evaluated for each data point.

### Protein Melting Temperature (T_m_) Measurements using Circular Dichroism spectroscopy

Circular Dichroism (CD) spectroscopy studies were carried out on a Chirascan-auto qCD machine (Applied Photophysics, USA) equipped with a qBiC Biocomparability Suite (Applied Photophysics, USA), using Dynamic Multi-mode Spectroscopy (DMS). Heat-induced denaturation of PhaP_*Ah*_ was monitored from 30 °C to 100 °C at a heating rate of 1 °C /min. The wavelength of 220 nm was used to draw the thermal graphs. Melting temperatures were calculated using the Global 3 software (Applied Photophysics, UK). An S curve was used to fit the thermal curves to the following equation^42^:


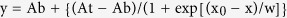


where by x_0_ refers to the melting temperature of a protein.

CD studies were performed in a buffer containing 50 mM Tris (pH 8.0) and 500 mM NaCl containing 0.5 mg/ml of wild-type or mutant protein, respectively.

### Fluorescence Spectroscopy

All fluorescence spectra were recorded on an F-2500 fluorescence spectrophotometer. The slit width was 5 nm for both excitation and emission. The excitation wavelengths were 280 nm and 380 nm for the intrinsic Tyr fluorescence and extrinsic ANS fluorescence, respectively. The extrinsic probe 8-Anilinonaphthalene-1-sulfonic acid (ANS) was added to the protein solutions at a molar ratio of 75:1 (probe:protein) and incubated for 30 min in the dark at 25 °C before measurement.

### Determination of Water-Oil Interfacial Tension - Pendant Drop Method

The interfacial tension between water (PhaP protein solution) and soybean oil was determined using an optical contact angle measurement and contour analysis system (DataPhysics, Germany). The setup was used to capture an image of a liquid drop that hangs on a dosing needle and to subsequently analyze it with the DataPhysics SCA 22 software module (DataPhysics, Germany) using the pendant drop method^43^.

### Determination of Surface Tension

Surface tension was measured based on the ring detachment method^44^ using a JZ-200A automated tensiometer (Chengde Precision Instruments Co. Ltd., Hebei, China) at 20 °C. The platinum ring was thoroughly rinsed with acetone and dried at 40 °C before each measurement. All samples were measured at the same protein concentration.

### Dynamic Light Scattering (DLS)

The molecular mass of PhaP_*Ah*_ in an aqueous buffer or in an emulsion containing 10 mM sodium oleate were analyzed using DLS recorded by a DynaPro Plate Reader II (Wyatt Technology, USA). The concentration of wild-type or mutant PhaP_*Ah*_ in the analytes was 0.5 mg/ml. The molecular mass was calculated using the DYNAMICS software suite (Wyatt Technology, USA).

### Static Light Scattering (SLS)

The molecular masses of full-length and N-terminally truncated PhaP_*Ah*_ in solution were determined via SLS using a DAWN HELEOSTM II 18-angle static light-scattering system (Wyatt Technology, USA) connected to a gel-filtration chromatography system using a Superdex 200 high performance column (GE Healthcare). The system was pre-equilibrated with buffer containing 50 mM Tris (pH 8.0) and 500 mM NaCl for more than 8 h. The pre-equilibrated system was then calibrated with BSA at a concentration of 1 mg/ml. Samples of full-length and N-terminally truncated PhaP_*Ah*_ were prepared as described above and concentrated to 1 mg/ml. Samples were injected into the SLS analyzer at a flow rate of 0.5 mL/min at 16 °C. The molecular mass was calculated using ASTRA5.3.4.14 software (Wyatt Technology, USA).

### Water Contact Angle Measurements

An OCA20 contact angle evaluation system (DataPhysics, Germany) was used to measure the water contact angles of drops of protein solution placed on the sample films. Droplets comprising 3 μL of deionized water with or without protein were carefully placed onto surfaces of poly (3-hydroxybutyrate-co-3-hydroxyhexanoate) or PHBHHx, and the average contact angle was obtained by measuring at least three different positions on the same PHBHHx film sample.

## Additional Information

**Accession codes:** The coordinates and structural factors have been deposited into the RCSB Protein Data Bank under the PDB accession code 5IP0.

**How to cite this article**: Zhao, H. *et al*. Structural Insights on PHA Binding Protein PhaP from *Aeromonas hydrophila. Sci. Rep.* **6**, 39424; doi: 10.1038/srep39424 (2016).

**Publisher's note:** Springer Nature remains neutral with regard to jurisdictional claims in published maps and institutional affiliations.

## Supplementary Material

Supplementary Information

## Figures and Tables

**Table 1:** Crystallographic data collection and refinement statistics.

**Data collection**	
Beamline	SSRF BL17U
Wavelength	0.9796 Å
Space group	C 2 2 2_1_
Cell dimensions	
a, b, c (Å)	131.79.174.14.198.82
α, β, γ (°)	90, 90, 90
Resolution (Å)	50.00–3.00 (3.07–3.00)
*R*_merge_ (%)	9.4 (86.3)
I/σI	15.5 (1.6)
Completeness (%)	99.7 (99.8)
Redundancy	5.3
**Refinement**	
Resolution (Å)	46.85–3.00 (3.07–3.00)
No. Reflections	43883
*R*_work_/*R*_free_ (%)	22.7/29.5
No. atoms	
Protein	12029
Cd cation	29
B-factor (Å^2^)	
Protein	113.7
Cd cation	207.8
r.m.s. deviations	
Bond lengths (Å)	0.014
Bond angles (°)	1.53
Ramachandran plot (%)	
Most favored	96.6
Additionally allowed	2.2
Generously allowed	0.6
Disallowed	0.6

R_*work*_ and R_*free*_ are defined as:

R = Σ hkl||Fobs| − |Fcalc||/Σ hkl|Fobs|, where h, k, and l are the indices of the reflections.

**Table 2:** The residues of hydrophobic and hydrophilic surface in the type I (Chains A and D) and II (Chains B and C) conformation.

Chain Type	Hydrophobic/Hydrophilic	Residues
**Type I**	Hydrophobic	A17, L20, L27, I31, L34, L37, A42, L48, L50, L53, V56, V59, L65, L68, V71, A76, L79, M83, L84, I87, L90, L93, F97, L101 L104
	Hydrophilic	T21, R22, Q25, S29, E32, Q33, R36, E47, Q54, K58, D61, T62, S64, T70, E74, T75, Q78, R81, D85, Q88, K89, Q95, Q96, E99, E100 D107
**Type II**	Hydrophobic	F9, M13, F16, L20, L27, I31, L34, L37, A42, L48, L50, L65, L68, L73, A76, L79, M83, L84, I87, L90, F97, L101, L104
	Hydrophilic	E11, Q14, T21, R22, Q25, S29, E32, Q33, R36, E47, Q63, S64, T70, E74, T75, Q78, R81, Q82, D85, D86, K89, Q96, E99 E100
