# Supplementary material for: Structural Insights on PHA Binding Protein PhaP from Aeromonas hydrophila
Source: Sci Rep. 2016 Dec 23;6:39424. doi: 10.1038/srep39424 (PMC5180188; doi:10.1038/srep39424)

Structural Insights on PHA Binding Protein PhaP from *Aeromonas hydrophila*

Authors: Hongyu ZHAO*[a], Hui WEI*[f], Xi LIU[b], Zhenyu YAO[a], Manyu XU[b], Daixu WEI[a], Jiawei WANG[#b], Xinquan WANG[#b,c], Guo-Qiang CHEN[#a,d,e]

Affiliations:

[a] Center for Synthetic and Systems Biology, School of Life Science, Tsinghua-Peking Center for Life Sciences, Tsinghua University, Beijing 100084, China
[b:] MOE Laboratory of Protein Science, Beijing Advanced Innovation Center for Structural Biology, Collaborative Innovation Center for Biotherapy, School of Life Sciences, Tsinghua University, Beijing 100084, China
[c:] Collaborative Innovation Center for Biotherapy, State Key Laboratory of Biotherapy and Cancer Center, West China Hospital, West China Medical School, Sichuan University, Chengdu, China

[d:] Center for Nano and Micro-Mechanics, Tsinghua University, Beijing 100084, China
[e:] MOE Key Lab for Industrial Biocatalysis, Tsinghua University, Beijing 100084
[f:] Key Laboratory of Cancer Prevention and Therapy National Clinical Research Center for Cancer, Tianjin Medical University Cancer Institute and Hospital, Tianjin, China

[*]Equal contribution

[#] Corresponding authors:

Guo-Qiang CHEN (Chen GQ)
School of Life Sciences, Tsinghua University, Beijing 100084, China
Phone: +86-10-62783844; Fax: +86-10-62794217
E-mail: chengq@mail.tsinghua.edu.cn

Xinquan WANG (WANG XQ)
School of Life Sciences, Tsinghua University, Beijing 100084, China
E-mail: xinquanwang@mail.tsinghua.edu.cn

Jiawei Wang (WANG JW)
School of Life Sciences, Tsinghua University, Beijing 100084, China
Email: jwwang@mail.tsinghua.edu.cn

Fig.S1. Gel filtration profiles and SDS-PAGE gels of purified whole length PhaP$_{Ah}$ and N-terminal truncation PhaP$_{Ah}$. The coordinate graph on the top right of each figure is a plot of log molecular weight against elution volume of the column. Gel filtration standards (Bio-Rad) used in the experiment were vitamin B12 (1.3 kD), horse myoglobin (17 kD),chicken ovalbumin (44 kD), bovine gamma-globulin (158 kD), and bovine thyroglobulin (670 kD). Calculated molecular weight from gel filtration of whole length PhaP$_{Ah}$ and N-terminal truncation PhaP$_{Ah}$ is 34.980 and 10.814 kD. However, the plots which Lg values were below 4 couldn't fit the line well. Static Light Scattering (SLS) have been done to measure the exact molecular weight of whole length PhaP$_{Ah}$ and N-terminal truncation PhaP$_{Ah}$ (Fig. S3).

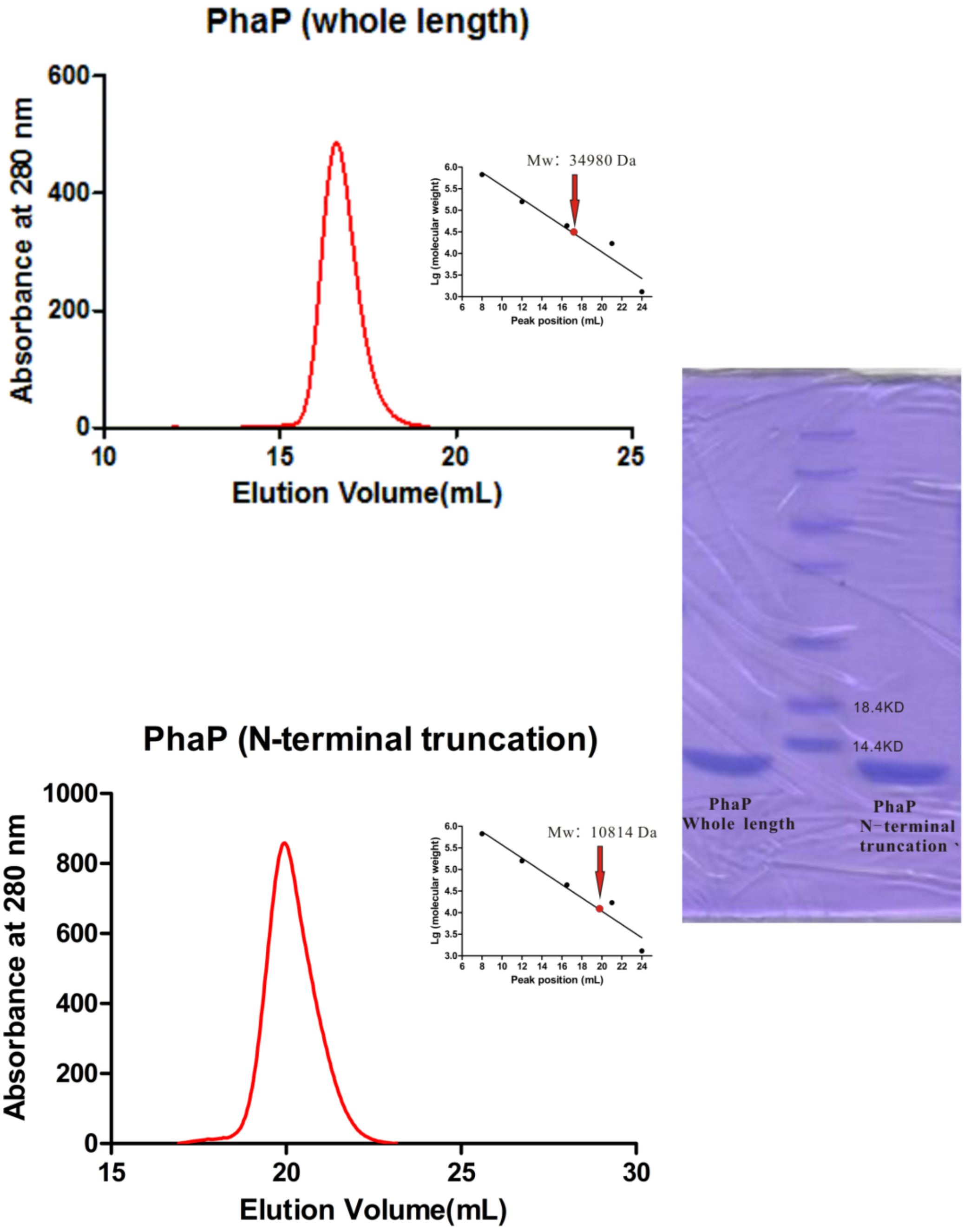

Fig. S2. DLS measurements of 0.5 mg/ml PhaP$_{Ah}$ sample in aqueous phase (A) and sodium oleate phase(B).

A

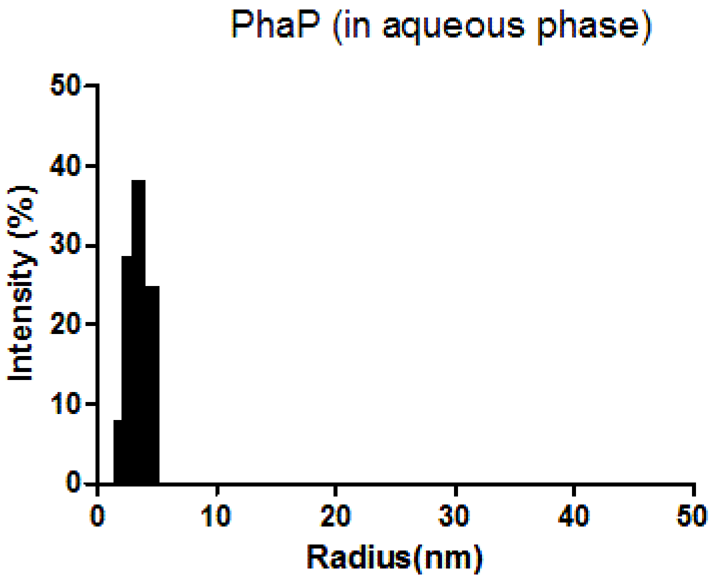

Buffer	Radius(nm)	Pd(%)	Mw-R(kDa)
50mM Tris Buffer	3.4	22.5	69

B

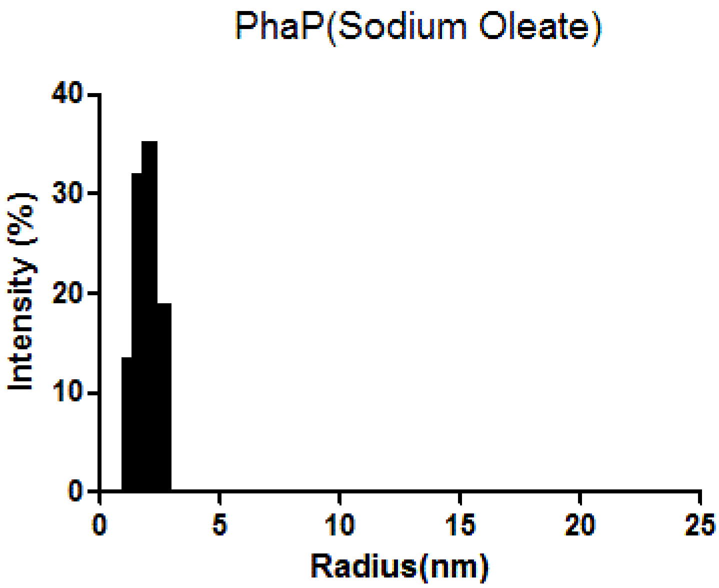

Buffer	Radius(nm)	Pd(%)	Mw-R(kDa)
10mM sodium oleate,50mM Tris Buffer	1.93	24.0	16

Fig. S3. SLS measurements of purified whole length PhaP$_{Ah}$ and N-terminal truncation PhaP$_{Ah}$

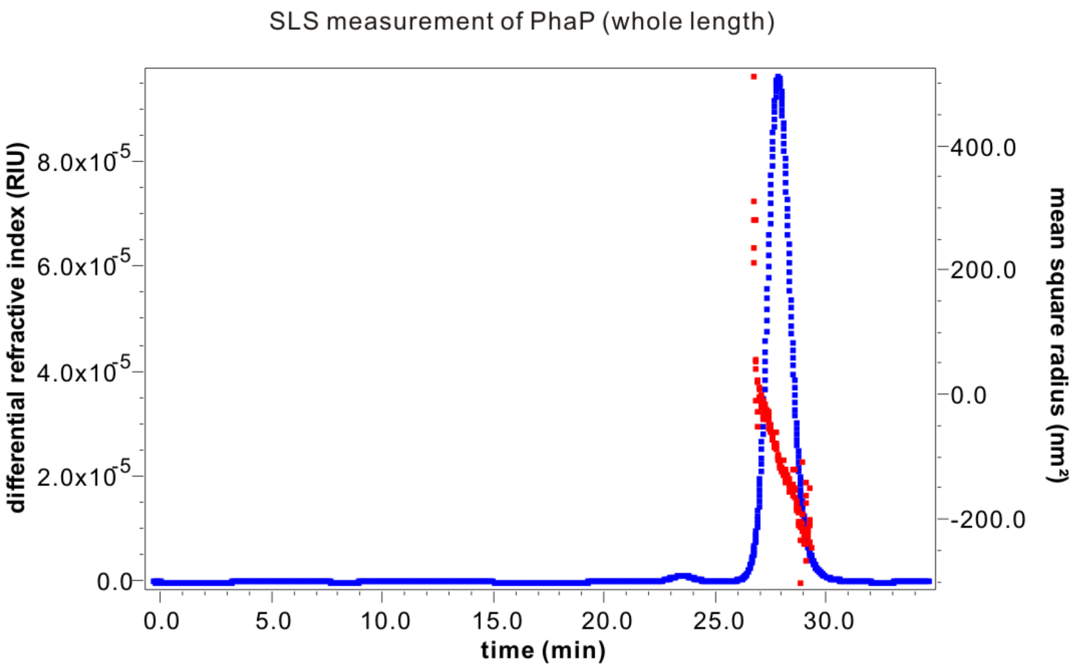

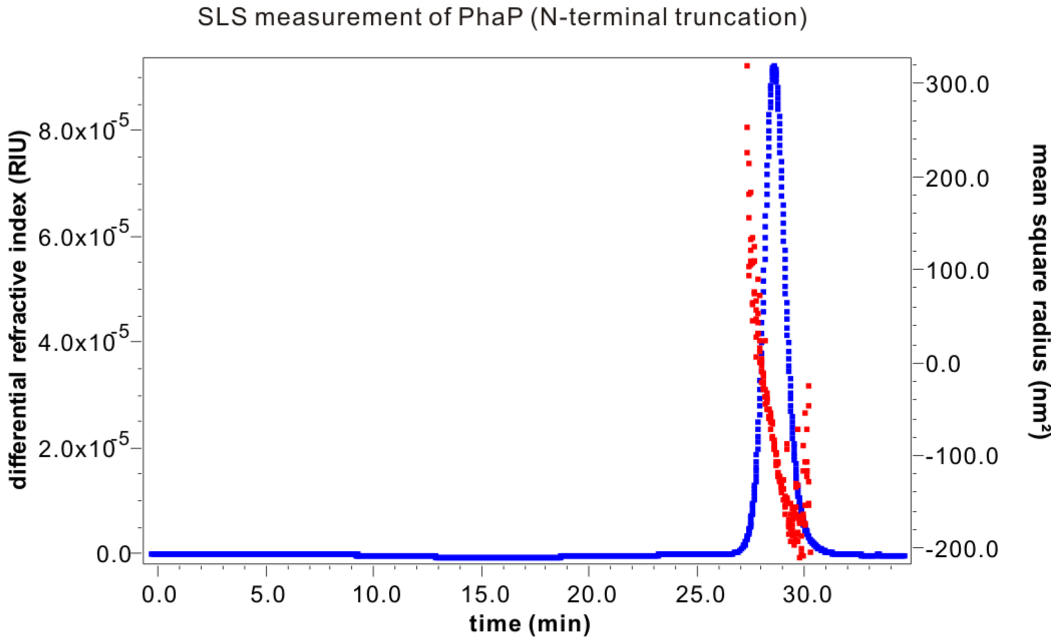

	**PhaP（whole length）**	**PhaP（N-terminal truncation）**
**Calculated Mass (μg)**	320.71	301.86
**Mn（Da）**	4.370×10^4	3.805×10^4
**Mp（Da）**	4.561×10^4	3.984×10^4
**Mw（Da）**	4.388×10^4	3.826×10^4

Fig. S4. Packing of in the space and interaction between each tetramer

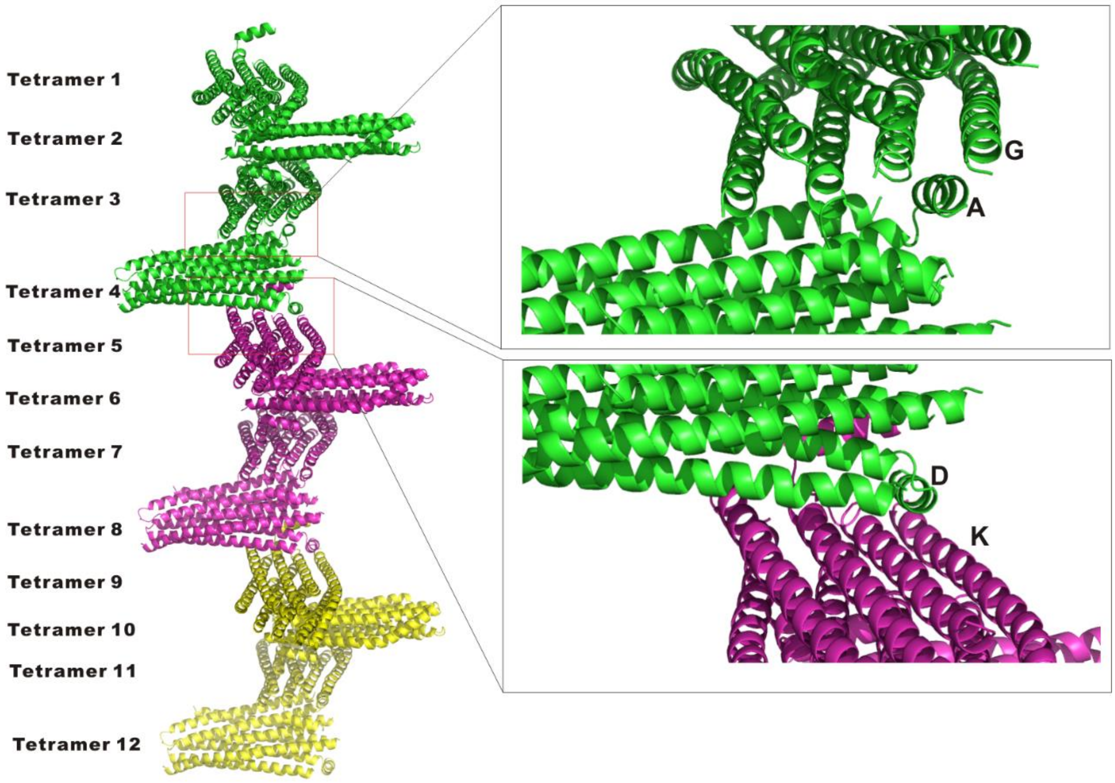

Fig. S5. N-terminal tail and the positive groove of the tetramer

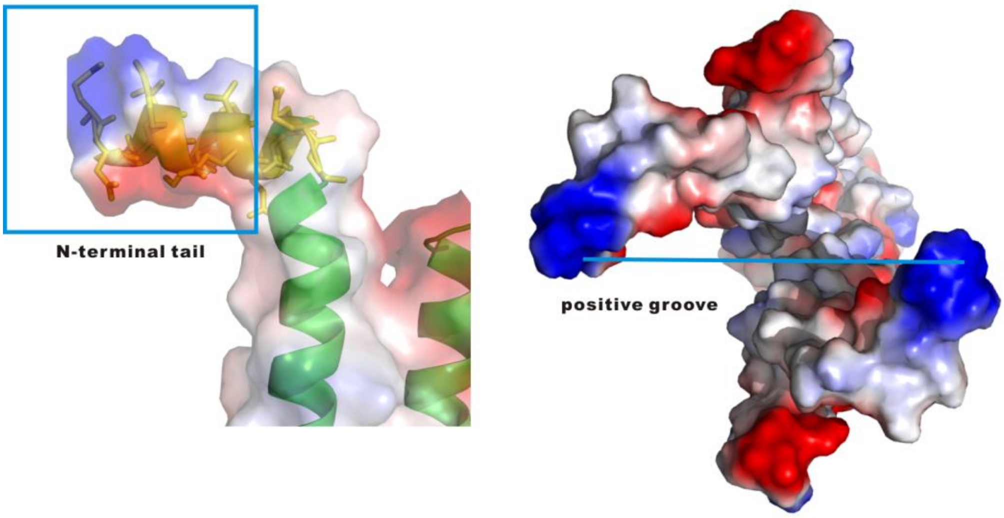

Fig. S6. Tyr residues has been labelled in the structure. All these Tyr residues are in the hydrophobic core which are inside the tetramer.

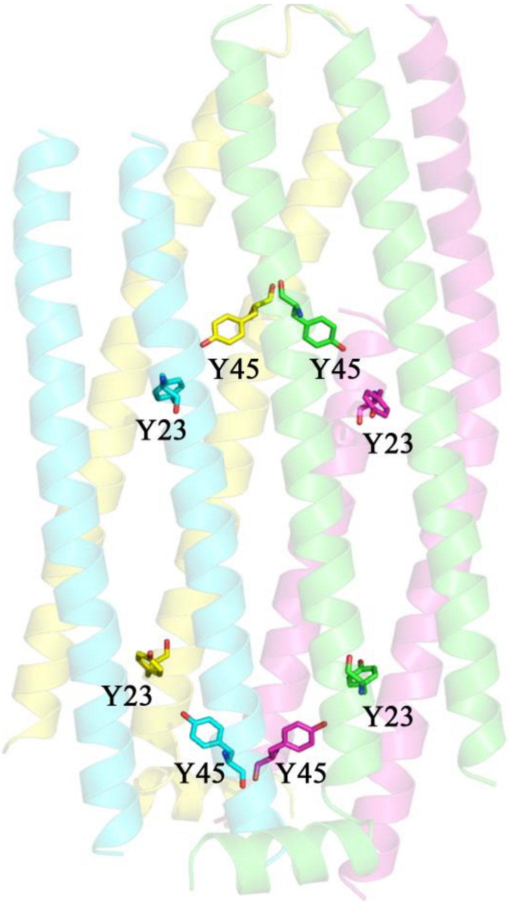

Fig. S7. Circular dichroism measurements of $PhaP_{Ah}$ and the buffer control (50 mM Tris and 500 mM NaCl)

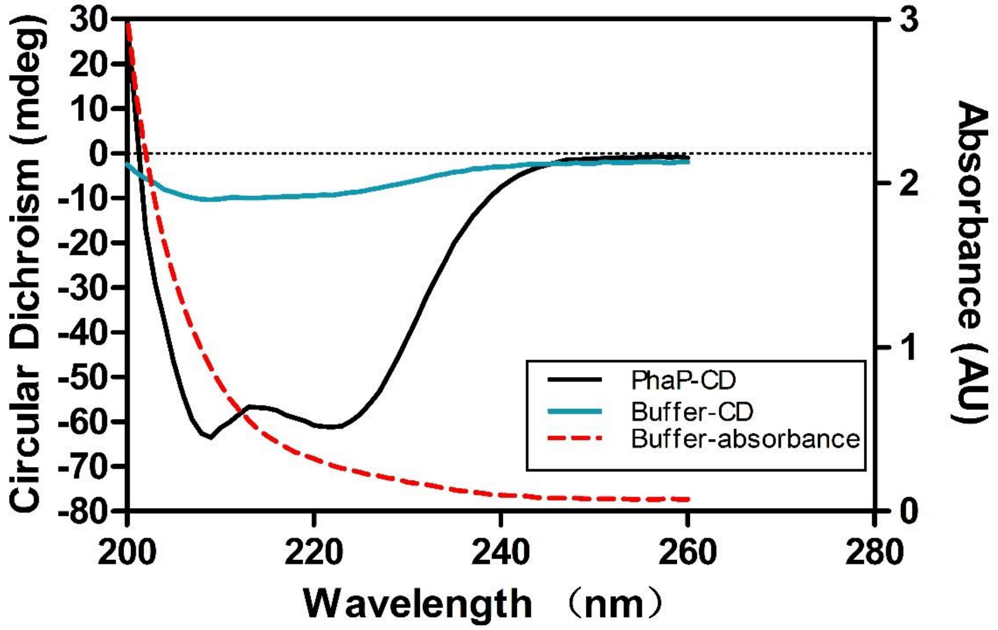

Supplement: Supplementary Information [file srep39424-s1.pdf]
